# PTEN loss mediated Akt activation promotes prostate tumor growth and metastasis via CXCL12/CXCR4 signaling

**DOI:** 10.1186/1476-4598-12-85

**Published:** 2013-07-31

**Authors:** M Katie Conley-LaComb, Allen Saliganan, Pridvi Kandagatla, Yong Q Chen, Michael L Cher, Sreenivasa R Chinni

**Affiliations:** 1 Departments of Urology and Pathology, Wayne State University School of Medicine, 9245 Scott Hall 540 E. Canfield Avenue, Detroit, MI 48201, USA; 2 The Barbara Ann Karmanos Cancer Institute, Wayne State University, Detroit, MI 48201, USA; 3 Department of Cancer Biology, Wake Forest University, Winston-Salem, NC 27157, USA

## Abstract

**Introduction:**

The chemokine CXCL12, also known as SDF-1, and its receptor, CXCR4, are overexpressed in prostate cancers and in animal models of prostate-specific PTEN deletion, but their regulation is poorly understood. Loss of the tumor suppressor PTEN (phosphatase and tensin homolog) is frequently observed in cancer, resulting in the deregulation of cell survival, growth, and proliferation. We hypothesize that loss of PTEN and subsequent activation of Akt, frequent occurrences in prostate cancer, regulate the CXCL12/CXCR4 signaling axis in tumor growth and bone metastasis.

**Methods:**

Murine prostate epithelial cells from PTEN^+/+^, PTEN^*+/−*^, and PTEN^−/−^ (prostate specific knockdown) mice as well as human prostate cancer cell lines C4-2B, PC3, and DU145 were used in gene expression and invasion studies with Akt inhibition. Additionally, HA-tagged Akt1 was overexpressed in DU145, and tumor growth in subcutaneous and intra-tibia bone metastasis models were analyzed.

**Results:**

Loss of PTEN resulted in increased expression of CXCR4 and CXCL12 and Akt inhibition reversed expression and cellular invasion. These results suggest that loss of PTEN may play a key role in the regulation of this chemokine activity in prostate cancer. Overexpression of Akt1 in DU145 resulted in increased CXCR4 expression, as well as increased proliferation and cell cycle progression. Subcutaneous injection of these cells also resulted in increased tumor growth as compared to neo controls. Akt1 overexpression reversed the osteosclerotic phenotype associated with DU145 cells to an osteolytic phenotype and enhanced intra-osseous tumor growth.

**Conclusions:**

These results suggest the basis for activation of CXCL12 signaling through CXCR4 in prostate cancer driven by the loss of PTEN and subsequent activation of Akt. Akt1-associated CXCL12/CXCR4 signaling promotes tumor growth, suggesting that Akt inhibitors may potentially be employed as anticancer agents to target expansion of PC bone metastases.

## Introduction

Chemokines are a superfamily of cytokines known to regulate the migration of cells and play a key role in the regulation of metastasis. The chemokine CXCL12, also known as stromal-derived factor-1 (SDF-1), is a potent chemoattractant for hematopoetic cells [1] and activate signaling events through its two distinct receptors, CXCR4 and CXCR7. CXCR4 has been shown to be a key receptor in mediating the metastasis of multiple types of tumors. Binding of CXCL12 to CXCR4 induces trimeric G protein signaling leading to activation of the Src, PI3K/Akt, ERK, and JNK pathways, contributing to protease production and cellular migration and invasion. In addition, we recently found that epidermal growth factor receptor family members are activated downstream of CXCL12/CXCR4 signaling, providing proliferative signals in bone tumor growth. CXCL12 and its receptors have been strongly linked to prostate cancer bone metastasis and are markers for poor prognosis [2-5].

The tumor suppressor phosphatase and tensin homologue deleted on chromosome 10 (PTEN, also known as MMAC1/TEP1) is a lipid and protein phosphatase that serves as a negative regulator of the phosphatidylinositol-3 kinase (PI3K) pathway [6]. PTEN dephosphorylates phos-phatidylinositol-3,4,5-trisphosphate (PIP3), thus serving as an inhibitor of the PI3K signaling pathway. Through its attenuation of the PI3K pathway, PTEN is a critical regulator of growth factor signaling and is able to regulate key cellular processes such as cell proliferation, motility, protein synthesis, glucose metabolism, genomic stability, and survival. Mutations of PTEN are associated with several diseases, including Cowden disease, Lhermitte-Duclos disease, and Bannayan-Zonana syndrome [7,8]. In addition, loss of PTEN has been shown to be associated with many types of cancer, such as glioblastoma, endometrial carcinoma, and breast cancer [9-11]. PTEN expression is frequently altered in cancer; PTEN is lost or mutated in 50-80% of primary PC, and complete loss of PTEN is associated with aggressive and metastatic cancer [5,12]. In mouse models of PC, loss of PTEN is critical for tumor initiation, and the level of PTEN expression is inversely associated with prostate tumorigenesis [13,14]. As shown by microarray analysis and immunohistochemistry, murine epithelial cells from these PTEN-deficient prostate tumors display increased expression of CXCL12 and CXCR4 as compared to the normal prostate glands of PTEN^+/+^ and PTEN^+/−^ mice [15]. However, it is not known whether Akt activation downstream of PTEN loss regulates CXCL12/CXCR4 expression and function in tumor cells.

In this study, by using cell lines derived from PTEN^+/+^, PTEN^+/−^, and PTEN^−/−^ mice, we demonstrate that loss of PTEN results in increased expression of CXCL12 and CXCR4. Using inhibitor assays, we demonstrate that regulation of the PI3K/Akt pathway by PTEN in turn regulates expression of both CXCL12 and CXCR4 in mouse and human prostate cancer cells. Akt overexpression in PTEN wild type DU145 cells induced cell proliferation, tumor growth and bone metastasis. Taken together, these data define a relationship between PTEN loss and CXCL12/CXCR4 signaling in prostate cancer progression.

## Materials and methods

### Cell culture

Cell lines were cultured in a humidified incubator with 5% CO2 at 37°C. All media were supplemented with 2 mM glutamine, 100units/ml penicillin, and 100 mg/ml streptomycin (Life Technologies Inc., Carlsbad, CA). Murine cell lines were maintained in Advanced DMEM supplemented with 5% fetal bovine serum. The benign human prostate cell line BPH-1 and human PC cell line PC3 were maintained in RPMI-1640 supplemented with 10% fetal bovine serum. Human PC cell line C4-2B and DU145 were maintained in T-Medium supplemented with 10% fetal bovine serum and DMEM supplemented with 10% fetal bovine serum, respectively.

### Establishment of PTEN^+/+^, PTEN^+/−^, and PTEN^−/−^ mouse prostate epithelial cell lines, DU145-neo and DU145-HA-Akt1 stable cell lines

Murine cell lines were established as described previously [16,17]. Briefly, exon 5 of PTEN was deleted specifically in the murine prostate. PTEN^+/+^, PTEN^+/−^, and PTEN^−/−^ prostate epithelial cells were isolated from prostates of corresponding mice at 8 weeks of age, and cell lines were established by serial dilution method and subsequent clonal selection. DU145 cells were transfected with PLNCX-neo and PLNCX- Hemagglutinin-tagged Akt1 constructs using lipofectamine 2000; 48 hours post-transfection, cells were exposed to Neomycin, and stable clones were selected.

### Western blot analysis

Cells were washed with PBS, and total cellular proteins were extracted with buffer containing 62.5 mM Tris–HCl (pH 6.8), 2% SDS, 1 mM PMSF, and 1X Protease inhibitor cocktail (Roche, Indianapolis, IN). Protein content was quantified with a BCA protein assay (Pierce Biotechnology, Inc, Rockford, IL), and equal amounts of protein were resolved by 10% SDS-PAGE. Immunoblot was performed with antibodies against PTEN, phosphorylated Akt (S473), and total Akt (Cell Signaling Technology, Boston, MA), CXCR4 (Chemicon, Billerica, MA) and GAPDH (Santa Cruz Biotechnology, Santa Cruz, CA). The band intensities were determined by quantitation of pixel intensities using ImageJ software (version 10.2; National Institutes of Health, Bethesda, MD).

### Quantitative RT-PCR

mRNA was purified from cells using the RNeasy kit (Qiagen, Valencia, CA), and cDNA synthesis was performed with iScript Select cDNA Synthesis Kit (Biorad, Hercules, CA). Real time RT-PCR was performed using SYBR Green mix plus ROX (Fisher Scientific) and the Eppendorf Mastercycler ep realplex^2^ qPCR System (Hauppauge, NY) according to the manufacturer’s protocol. Relative values of gene expression were normalized to GAPDH and calculated using the 2^-ΔΔCt^ method, where ΔΔCt = (ΔCt_target gene_ - ΔCt_GAPDH_)_sample_ - (ΔCt_target gene_ - ΔCt_GAPDH_)_control_. The fold change in relative expression was then determined by calculating 2^-ΔΔCt^. Forward and reverse murine specific primers used are as follows: CXCR4: 5′-TCAGTGGCTGACCTCCTCTT-3′, 5′-TTTCAGCCAGCAGTTTCCTT-3′; CXCL12: 5′-CTTCATCCCCATTCTCCTCA-3′, 5′-GACTCTGCTCTGGTGGAAGG-3′. Forward and reverse human specific primers used are as follows: CXCR4: 5′-GGTGGTCTATGTTGGCGTCT-3′, 5′-TGGAGTGTGACAGCTTGGAG-3′; CXCL12: 5′-ATGAACGCCAAGGTCGTG-3′, 5′-CTTCGGGTCAATGCACACTT-3′. Forward and reverse primers recognizing both murine and human GAPDH were 5′- ATCACCATCTTCCAGGAGCGA-3′ and 5′-GCCAGTGAGCTTCCCGTTCA-3′, respectively.

### Inhibition of Akt signaling pathway

Cells were cultured with growth media supplemented with 1% FBS and treated with indicated concentrations of Akt Inhibitor IV (Fisher Scientific, Pittsburgh, PA) or vehicle control for 18 hours. Subsequently, mRNA and protein were collected from cells and subjected to quantitative RT-PCR analyses or western blot analysis, respectively.

### Invasion assay

Cells were cultured with complete growth media and treated with 10 μM Akt Inhibitor IV or vehicle control; after five hours, media was replaced with growth media supplemented with 1% FBS containing 10 μM Akt Inhibitor IV or vehicle control. After overnight culture, cells were trypsinized and plated on the upper chamber of matrigel-coated transwell filters (2x10^5^ cells/filter) in growth media supplemented with 1% FBS containing 10 μM Akt Inhibitor IV or vehicle control, with 200 ng/mL CXCL12 added to bottom chamber. After 24 hours, cotton swabs were used to remove unmigrated cells from the upper chamber, and inserts were stained with 0.9% crystal violet. Total number of migrated cells was counted under 10× magnification. Assay was performed in triplicate.

### *In vivo* studies and tumor tissue analyses

Both subcutaneous and intratibial tumor inoculation studies were performed as described previously [18]. Briefly, five-week-old male C.B.-17 severe combined immunodeficient (SCID) mice (Taconic Farms, Germantown, NY) were used in the study. For subcutaneous tumor cell implantation, 5x10^5^ cells of both DU145-Neo and DU145- Hemagglutinin-tagged AKT1 transfectants were mixed in 50% matrigel in a volume of 100 μl and implanted in flanks of mice. For each cell line, eight mice were used in the experiment. For intratibial implantation 1x10^5^ cells were injected per bone; for each cell line, 8–10 mice were used. Histomorphometric analyses were performed to determine tumor burden and trabecular bone area in both DU145 transfectants (Neo and HA-Akt1) as previously described [18].

### Immunohistochemistry

Formalin-fixed, paraffin-embedded serial tissue sections from DU145-Neo and DU145-HA-Akt1 tumors were deparaffinized with xylene and rehydrated in graded EtOH. Endogenous peroxidase activity was blocked by incubating in 3% H_2_O_2_ for 20 min. For subcutaneous tumor sections, antigen retrieval was performed with An-tigen Retrieval Citra Plus Solution (BioGenex, Freemont, CA) in a steamer. For bone sections, antigen retrieval was performed with proteinase K (Sigma-Aldrich, St. Louis, MO). Slides were then blocked with Blocking Serum from ABC Vectastain Kit (Vector Labs, Burlin-game, CA). Slides were incubated at 4°C overnight in a humidified chamber with antibodies directed against Ki67 (BD Biosciences, San Jose, CA), phosphorylated Akt (S473) (Cell Signaling Technology), or CXCR4 (R&D Systems, Minneapolis, MN). After washing, sections were incubated with ABC Vectastain Kit, according to manufacturer’s protocol, followed by incubation with 3,3-diaminobenzidine tetrahydrochloride (Vector Labs). Nuclei were counterstained with Mayer’s hematoxylin (Sigma-Aldrich). Sections were then dehydrated with graded EtOH, washed with xylene, and mounted with Permount (Sigma-Aldrich).

### Statistical analyses

Data were analyzed using Microsoft Excel 2008. All data are presented as mean ± SD. Data were analyzed using Student’s t-test; a p-value < 0.05 was considered statistically significant.

## Results

### Progressive loss of PTEN results in increased expression of CXCL12 and CXCR4 in murine prostate epithelial cells

**Figure 1:**
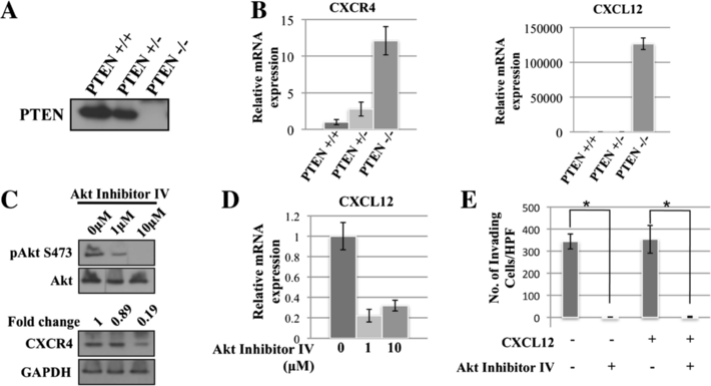
**Loss of PTEN results in increased expression of CXCL12 and CXCR4 in murine prostate epithelial cells. A)** Cell lysates were collected from PTEN^+/+^, PTEN^+/−^, and PTEN^−/−^ cells and analyzed by Western blot for PTEN. **B)** mRNA expression levels of CXCL12 and its receptor, CXCR4, in PTEN^+/+^, PTEN^+/−^, and PTEN^−/−^ cells were analyzed by quantitative RT-PCR. **C**, **D)** PTEN^−/−^ cells were treated for 18 hours with increasing concentrations of Akt Inhibitor IV. Protein and mRNA expression levels were analyzed by Western blot **(C)** and quantitative RT-PCR **(D)**, respectively. **E)** PTEN^−/−^ cells were pretreated with or without 10 μM Akt Inhibitor IV; 2x10^5^ cells were then plated on Matrigel coated inserts, allowed to invade for 24 hours, and stained with Crystal Violet. Total number of migrated cells was counted under 10X magnification in five fields. Assay was performed in triplicate. *: p < 0.005.

In an effort to study the regulation of CXCL12 and its receptor, a murine model was utilized. In this model system, prostate epithelial cell lines were generated from anterior prostates of Pten^+/+^, Pten^+/−^, and Pten^−/−^ mice, using the method previously described [15,16,19]. Loss of PTEN was verified at the protein level (Figure 1A). As shown by qPCR, PTEN^−/−^ cells exhibit significantly increased mRNA levels of CXCL12 and CXCR4 (Figure 1B). These data are consistent with results from Berquin et al., where microarray and immunohistochemistry demonstrated increased expression of both CXCL12 and CXCR4 in PTEN^−/−^ mice [15]. When PTEN^−/−^ cells were treated with increasing concentrations of Akt Inhibitor IV, expression of both CXCR4 and CXCL12 decreased (Figure 1C,D). As expected, Akt Inhibitor IV inhibited Serine 473 phoshporylation on Akt without changing Akt1 levels in cells. As low as 1 μM Akt Inhibitor IV reduced Serine 473 phosphorylation. At this concentration, Akt Inhibitor IV abrogated basal as well as CXCL12-induced cell invasion of PTEN^−/−^ cells through Matrigel coated inserts (Figure 1E). Notably, there was no significant difference in cell invasion when 200 ng/mL CXCL12 was added to the bottom chamber, likely due to the high basal levels of CXCL12 expressed by PTEN^−/−^ cells.

### Akt regulates CXCR4 expression in PTEN-null human prostate cancer cells

**Figure 2:**
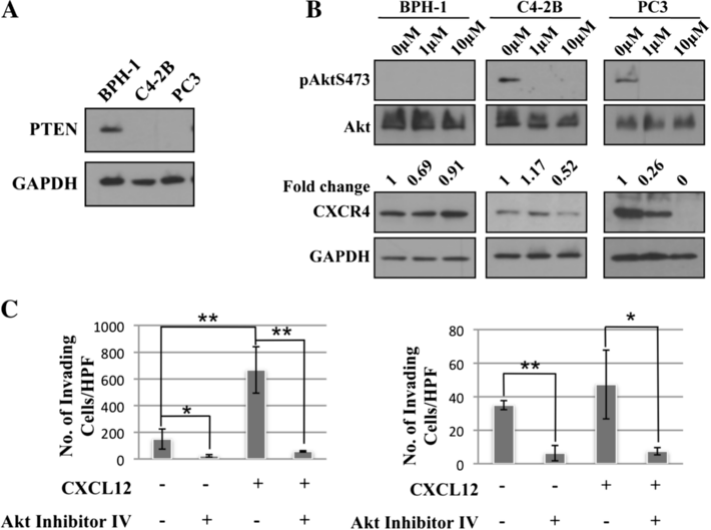
**Akt regulates CXCR4 expression in PTEN-null human prostate cells. A)** Cell lysate was collected from BPH-1, C4-2B, and PC3 cells. Protein levels of PTEN and β-actin were analyzed by Western blot. **B)** BPH-1, C4-2B, and PC3 cells were treated for 18 hours with increasing concentrations of Akt Inhibitor IV. Protein levels were analyzed by Western blot. **C)** C4-2B (*left*) and PC3 (*right*) cells were pretreated with or without 1 μM Akt Inhibitor IV; 2x10^5^ cells were then plated on Matrigel coated inserts, allowed to invade for 24 hours, and stained with Crystal Violet. Total number of migrated cells was counted under 10X magnification in five fields. Assay was performed in triplicate. *: p < 0.05; **: p < 0.015.

To examine the role of PTEN in the regulation of CXCR4 in human prostate cancer, the cell lines BPH-1, C4-2B, and PC3 were utilized. As shown in Figure 2A, BPH-1 expresses PTEN, while C4-2B and PC3 are PTEN-null. Treatment with 1 and 10 μM Akt Inhibitor IV resulted in decreased expression of CXCR4 in C4-2B and PC3 cell lines (Figure 2B). As low as 1 μM Akt Inhibitor IV reduced CXCR4 expression in PC-3 cells, whereas in C4-2B cells 10 μM Akt inhibitor IV inhibited CXCR4 expression. Additionally, CXCL12-mediated invasion through a matrigel-coated transwell insert was abrogated by treatment with 1 μM Akt Inhibitor IV in both C4-2B and PC3 (Figure 2C).

### Overexpression of Akt results in increased phosphorylation of Akt, CXCR4 expression, proliferation and invasion

**Figure 3:**
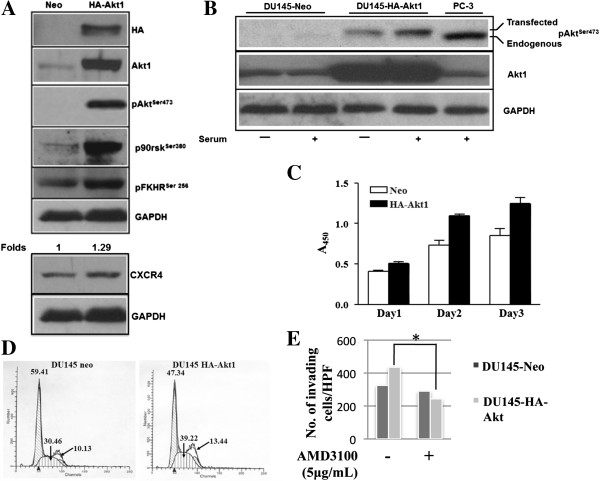
**Overexpression of Akt1 results in increased phosphorylation of Akt, CXCR4 expression, and proliferation. A)** DU145 cells were stably transfected with HA-tagged Akt1. Lysate was collected from serum-starved cells, and protein levels were analyzed by Western blot. **B)** Cells were cultured in the presence or absence of serum, lysate was collected, and protein levels were analyzed by Western blot. **C)** Proliferation of DU145-Neo and DU145-HA-Akt1 cells was determined over three days. **D)** Cell cycle analysis of DU145-Neo and DU145-HA-Akt1 cells was performed. **E)** DU145-Neo or DU145-HA-Akt1 cells treated with 5 μM AMD3100 for two hours were plated on Matrigel coated inserts, allowed to invade for 24 hours, and stained with Crystal Violet. Total number of migrated cells was counted under 10X magnification in five fields. *: p < 0.01.

Multiple cell surface receptors have been shown to activate Akt kinase and induce downstream signaling events leading to cell survival. Among Akt family members Akt1 is predominantly expressed in prostate cancer cells[20]. Even though PTEN lipid phosphatase activity has been shown to regulate the PI3K-Akt pathway, several studies document PI3K-Akt-independent functions of PTEN [21-23]. PTEN loss deregulates both lipid and protein phosphatase activity [24]. Figures 1 and 2 demonstrate that Akt activation regulates CXCR4 expression. To determine Akt1 function in tumor growth and metastasis without disturbing other functions of PTEN, a novel model consisting of Akt1 overexpression in PTEN-intact DU145 cells was generated. Studies have been performed previously using a constitutively active Akt via artificially tagging membrane localization myristoylation signal to study downstream functions of activated Akt; however, in these studies, the transfected Akt must be phosphorylated in the cell to induce downstream effects similar to endogenous Akt protein. DU145 cells transfected with HA-Akt1 exhibit increased levels of pAkt Ser473, p90rskSer380 and pFKHR Ser256 in serum free media, suggesting that transfected Akt1 and its effector signaling is activated in cells (Figure 3A). In addition, Akt1 overexpression induced a 1.29 fold increase in CXCR4 protein expression (Figure 3A). Culture of the cells with 10% serum resulted in a further increase of phosphorylated Akt (Figure 3B). When cells were cultured in complete serum conditions, HA-Akt1 expression resulted in an increase in proliferation compared to Neo-transfected cells (Figure 3C). Additionally, cell cycle analysis revealed that expression of HA-Akt1 resulted in a decrease in the G_0_G_1_ population and an increase in the S phase population (Figure 3D), suggesting cell cycle progression. To demonstrate that CXCR4 is a key element of Akt-induced effects, an invasion assay was performed utilizing AMD3100, a pharmacological inhibitor of CXCR4. DU145-HA-Akt1 cells exhibited increased invasion through Matrigel coated inserts, as compared to DU145-Neo cells (Figure 3E). Treatment of DU145-Neo cells with AMD3100 did not affect invasion. In DU145-HA-Akt1 cells, however, invasion was inhibited by this treatment, suggesting that the increased invasion of Akt1-transfected cells as compared to control cells is driven at least in part by CXCL12/CXCR4 signaling. These studies together demonstrate Akt1 activity in DU145 cells, and that this activity induces CXCR4 expression and function.

### Overexpression of Akt results in increased subcutaneous tumor growth

**Figure 4:**
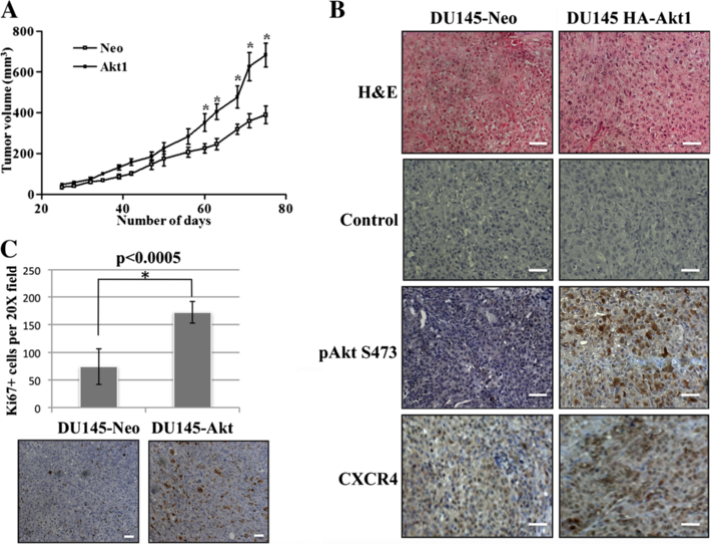
**Overexpression of Akt1 results in increased subcutaneous tumor growth.** DU145-Neo and DU145-HA-Akt1 cells were injected into mice subcutaneously. **A)** Tumor volume was measured over 80 days. **B)** Tissue sections from DU145-Neo and DU145-HA-Akt1 subcutaneous tumors were immunostained with antibodies directed against pAkt(S473) or CXCR4, or with negative control. Images were taken at 20X. Bar represents 50 μM. **C)** Proliferation of DU145-Neo and DU145-HA-Akt1 tumors was analyzed by Ki67 staining; average number of Ki67+ cells of five 20X fields was determined. Bar represents 50 μM.

To determine the biological importance for Akt in tumor growth, mice were injected subcutaneously with DU145-Neo or DU145-HA-Akt1 cells. As shown in Figure 4A, HA-Akt1 expression resulted in increased tumor volume after 60 days of inoculation; the growth rate was significantly faster compared to DU145-neo cells. As shown by immunohistochemistry, tumors also exhibited increased expression of both Serine 473 phosphorylated Akt and CXCR4, suggesting that activated Akt mediates downstream gene expression, resulting CXCR4 overexpression (Figure 4B). Furthermore, Ki67 staining revealed increased proliferation in DU145-HA-Akt1 tumors as compared to neo controls (Figure 4C). These data demonstrate that overexpressed Akt is active in tumors and mediate tumor growth by enhancing CXCR4 sig-naling.

### Overexpression of Akt1 results in increased intratibial tumor growth

**Figure 5:**
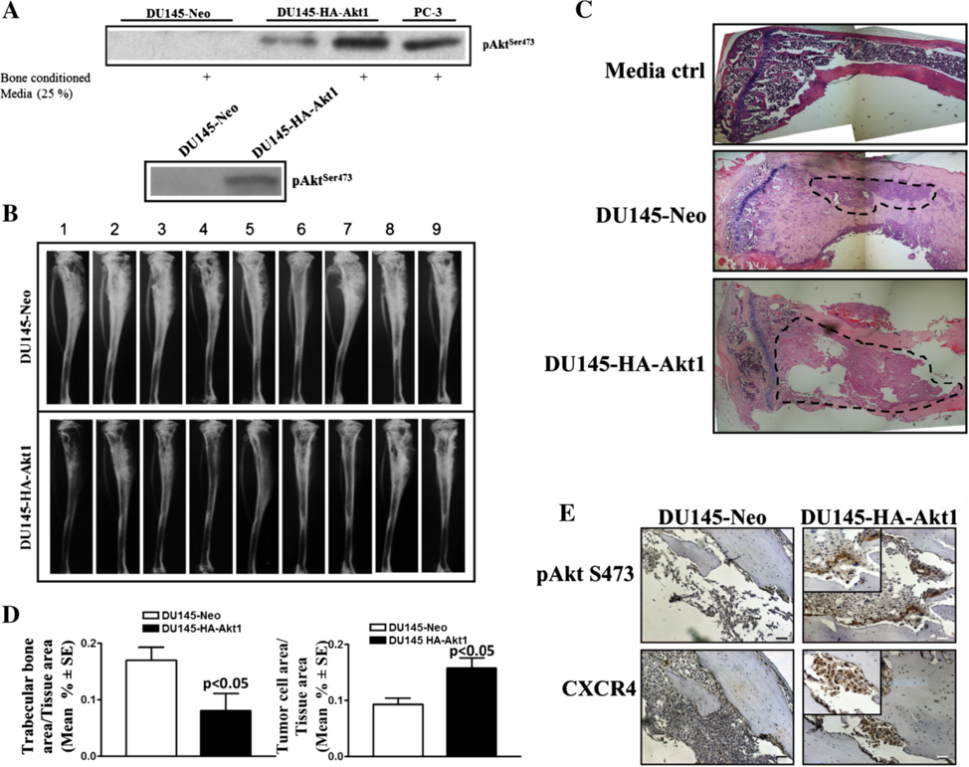
**Overexpression of Akt1 results in increased intratibial tumor growth. A)** Cells were cultured in presence or absence of bone conditioned medium (upper panel) and co-cultured with human bone stromal cells (lower panel), then analyzed by Western blot for pAkt(S473). **B)** Cells were injected into the tibiae of mice intratibially. After harvesting at 8 weeks, bones were analyzed by x-ray. **C)** H&E was performed on tissue sections from DU145‒Neo and DU145‒HA‒Akt1 intratibial tumors. Images were taken at 5X and digitally merged. **D)** Trabecular bone and tumor growth was analyzed at 8 weeks. **E)** Tissue sections from DU145-Neo and DU145-HA-Akt1 intratibial tumors were immunostained with antibodies directed against pAkt(S473) or CXCR4. Images were taken at 20X and 40X (insert). Bar represents 50 μM.

Prostate cancer frequently metastasizes to the bone, and previous studies implicate key role for CXCL12/CXCR4 signaling in bone metastasis. To examine the effects of Akt1 in the bone environment, DU145-HA-Akt1 cells were cultured with bone conditioned media, resulting in increased Serine 473 phosphorylation. This increase in phosphorylation was not detected in DU145-Neo control cells (Figure 5A). Further, co-culture of DU145 transfectants with human fetal bone stromal cells show that in HA-Akt1 transfected cells Akt is phosphorylated at Serine 473, suggesting that Akt signaling in cancer cells is induced by bone stromal interactions in both a paracrine manner and in direct contact. Next, mice were injected intratibially with DU145-Neo or DU145-HA-Akt1 cells. Previous studies show that DU145 cells in intratibial model induce an osteosclerotic phenotype, as evidenced by enhanced trabecular bone formation [18]. DU145-Neo cells induced a similar osteosclerotic reaction in bone, while DU145-HA-Akt1 cells resulted in increased osteolysis at eight weeks (Figure 5B,C). Histomorphometric analysis reveal that DU145-HA-Akt1 tumors exhibited a decreased ratio of trabecular bone area to tissue area, as well as an increase in overall tumor burden in bone tumors (Figure 5D). Furthermore, DU145-HA-Akt1 tumors expressed higher levels of phosphorylated Akt and CXCR4 (Figure 5E).

## Discussion

Chemokines and their receptors play key roles in hematopoietic cell trafficking. CXCL12/CXCR4 has also been shown to play a key role in the regulation of metastasis, and its expression has been shown to be elevated in localized and metastatic cancer, including bone metastatic prostate tumors [2,3]. Among PC patients, higher expression of CXCR4 was documented in prostate tumor tissues from African American patients, suggesting CXCR4 expression is associated with aggressive disease phenotypes in these patients [25]. Human prostate tumor expression of CXCR4 is also associate with poor survival [4], as its expression is significantly associated with local recurrence after therapy and formation of distant metastases [5]. Taken together, these studies emphasize the clinical significance of CXCL12/CXCR4 expression in prostate tumor progression.

Tumor cells expressing CXCR4 metastasize to target organs that express high levels of CXCL12 [26,27]. Colonization of tumor cells in the bone microenvironment is thought to occur through PC cell encroachment of the hematopoietic stem cell niche by mimicking the stem cell interactions with bone resident stromal cells [28]. This concept is validated by studies showing that targeting CXCR4 function through neutralizing antibodies inhibited prostate cancer bone metastasis [29], and overexpression of CXCR4 in prostate cancer cells enhanced bone metastasis [30]. Additionally, inhibition of CXCR4 with CTCE-9908 resulted in decreased tumor growth, angiogenesis, and lymphangiogenesis, as well as increased apoptosis in a xenograft PC model [31]. Thus, these studies show that the CXCL12/CXCR4 axis in tumor cells usurps stem cell homing mechanisms to get into bone [28,29] and subsequent colonization and growth through activation of growth factor receptor signaling [30]. Studies with colorectal cancers show that CXCR4 signaling is also involved in outgrowth of metastasis [32]. Taken together, these studies demonstrate that CXCL12/CXCR4 signaling is a critical event mediating homing of tumor cells and subsequent expansion of metastases.

Our previous studies show that PTEN knockout mouse model epithelial tumor cells gain expression of an “osteogenic signature,” thus predisposing these cells for metastasis [15]. We found that both CXCL12 and CXCR4 are overexpressed in epithelial tumor cells in PTEN knockout mice. Herein, using cultured cells from PTEN intact, heterozygous, and knockout cells, we show that both CXCR4 and CXCL12 expression is higher in PTEN knockout cells, thus confirming the immunohistochemical tumor expression findings (Figure 1). Specific inhibition of Akt resulted in downregulation of both CXCL12/CXCR4 expression, and an established PI3 kinase inhibitor also downregulated both CXCL12 and CXCR4 gene expression (data not shown). Similarly, Akt-mediated regulation of CXCR4 was observed in human prostate cancer cells with loss or mutation of PTEN (Figure 2). Additionally, in both mouse and human tumor cells with either loss or mutation of PTEN, Akt Inhibitor IV treatment inhibited basal as well as CXCL12-induced invasion. PTEN loss-induced PI3K/Akt has been shown to mediate migration and invasion of prostate cancer cells in response to CXCL12/CXCR4 [20,33,34], suggesting that Akt can function both upstream (as an inducer of CXCR4 expression) and downstream (as a signaling kinase for induction of proteases and invasion) of CXCR4. Current studies with Akt inhibitors implicate Akt as a key member in this pathway contributing to CXCR4 expression in prostate cancer cells.

Tumor suppressive functions of PTEN independent of phosphoinositide lipid phosphatase activity play a key role in cell cycle regulation, maintaining genomic instability, and controlling DNA repair mechanisms that safeguard cells from accumulation of genetic mutations and uncontrolled proliferation [24]. PTEN loss dysregulates these genome safeguard mechanisms and also leads to Akt activation, promoting tumorigeneisis. To determine the function of Akt in tumor growth and metastasis via regulation of CXCR4 function independent of other effects induced by PTEN loss, DU145 cells were transfected with an HA-tagged Akt1, which is not constitutively active and must be activated within the cell (Figure 3). Akt1 overexpression resulted in increased proliferation and cell cycle progression, suggesting that transfected Akt1 mimicked PTEN loss-associated hyperactivated Akt signaling. In an effort to determine the biological significance of Akt/CXCR4 axis in tumor growth, we subcutaneously implanted both low Akt1 (Neo) and high Akt1 (HA-Akt1) expressing cells in SCID mice (Figure 4). Akt1 overexpression significantly enhanced tumor growth starting from day 60. Immunohistochemical analysis further revealed that HA-Akt1 tumors proliferated at a faster rate, as shown by increased staining for Ki-67 proliferation marker. As expected, HA-Akt1 tumors have stronger Akt Serine 473 phosphorylation and CXCR4 expression, suggesting that Akt1 induced CXCR4 expression, contributing to the primary tumor growth. Our previous data demonstrate that CXCR4 overexpression in the PTEN-null cell line PC-3 enhanced bone tumor growth in a SCID-human model [20]; these tumors have activated Akt signaling, demonstrating the Akt signaling downstream of CXCR4 contributing to bone tumor growth. Herein, we utilized DU145-HA-Akt1 overexpressing cells to specifically determine the contribution of Akt1 without perturbing the lipid/protein phosphatase functions of PTEN in CXCR4 expression and downstream signaling in bone tumor growth. Activation of Akt1 in these cells was enhanced by bone factors and/or bone stromal cell interactions. In intratibial models, DU145 cells produce osteosclerotic reactions, as evidenced by enhanced bone formation measured by X-rays and histomorphometry. DU145-Neo cells produced similar bone reactions in our model, whereas overexpression of Akt1 completely reversed this phenotype to an osteolytic phenotype similar to PC-3 cells in this model, as evidenced by enhanced tumor growth and destruction of trabecular bone (Figure 5). Akt1 activation, as measured by Serine 473 phosphorylation, is higher in DU145-HA-Akt1 cells and is localized to the bone tumor interface, suggesting that transfected Akt1 is activated by bone remodeling-released and/or stromal-expressed factors. CXCR4 expression is also enhanced in bone tumors (Figure 5E), suggesting that CXCL12/CXCR4 signaling contributing to bone tumor growth in Akt1 transfected DU145 cells.

## Conclusion

In summary, these data showed that PTEN loss-induced PI3K/Akt pathway induces CXCL12/CXCR4 expression, and this expression is particularly mediated by Akt kinase in both murine and human prostate cancer cells. Akt1-induced CXCR4 expression is active in CXCL12-induced cellular invasion, tumor growth, and intraosseous tumor growth in murine model systems. Given the frequency of PTEN gene alterations in advanced human prostate tumors and expression of CXCR4 in these patients, Akt1 signaling may be a therapeutic target for advanced prostate cancer patients.

## Competing interests

The authors declare that they have no competing interests.

## Authors’ contributions

MKC-LC participated in study design, performing experiments, data analysis and drafting of manuscript. AS and PK performed in vivo experiments and data analysis. YQC participated in study design and PTEN knockout cell generation. MLC participated in study design and manuscript editing. SRC is involved in conception and design of study, data preparation and analysis, manuscript drafting and revisions. All authors read and approved the final manuscript.
